# Keratinocyte Capability for Smooth Sheet Formation on a Step Pattern Substrate

**DOI:** 10.3390/bioengineering12090929

**Published:** 2025-08-29

**Authors:** Nozomu Kitamura, Shoichi Makara, Masami Kojima, Isao Tsuyumoto, Masahiro Sokabe, Kennedy Omondi Okeyo, Hiroaki Hirata

**Affiliations:** 1 Department of Life Science and Biotechnology, Kanazawa Institute of Technology, Hakusan 924-0838, Ishikawa, Japan; 2 Department of Chemistry and Environmental Science, Kanazawa Institute of Technology, Nonoichi 921-8501, Ishikawa, Japan; 3 Human Information Systems Laboratories, Kanazawa Institute of Technology, Hakusan 924-0838, Ishikawa, Japan; 4 Weldon School of Biomedical Engineering, Purdue University, West Lafayette, IN 47907, USA

**Keywords:** keratinocyte, epidermis, tissue morphogenesis, adherens junction, cell proliferation, microfabrication

## Abstract

The proper architecture of the epidermis is crucial for the barrier function of the skin against the external environment. In interfollicular regions, the epidermis preserves a flat outer surface despite the undulating topography of the underlying basement membrane. However, the mechanisms by which the epidermis adapts its architecture to the basal topography remain unclear. In this study, using a substrate with micropatterned steps of a few cell heights, we demonstrate for the first time that keratinocytes on the topographically patterned substrate autonomously form a cell sheet with a smooth upper surface. Keratinocytes accumulate at higher densities near the rising edge of a step, resulting in a cell sheet with a logarithmic slope on its upper surface. Furthermore, we find that both adherens junction-mediated intercellular adhesion and cell proliferation are essential for the formation of the smooth keratinocyte sheet. These findings suggest the robustness of keratinocyte population morphogenesis against variations in basal topography, providing new insights into skin tissue engineering.

## 1. Introduction

The skin covers the outer surface of our body, serving as a protective barrier. The barrier function of the skin depends on the structural integrity of the skin tissue. The skin tissue consists of a layered structure: the outer layer, epidermis, and the inner layer, dermis, connected by the basement membrane [1]. In the epidermis, keratinocytes, the primary cells of the epidermis, form multiple layers called strata. Basal keratinocytes adhere to the basement membrane and proliferate. As they differentiate, they move toward outer layers, finally shedding from the body surface [1].

The epidermis in interfollicular regions contains prominent epidermal rete ridges, undulating microstructures that extend into the dermis [2,3]. The size of rete ridges in human skin ranges 50–400 μm in width and 10–200 μm in length, which depends on age, anatomical location, and ethnicity [2,4,5]. Dermal papillae, projections of the dermis into the epidermis, are perfectly embedded between the rete ridges [2,3]. Thus, the basement membrane as the epidermis–dermis boundary exhibits undulating topography, contributing to the stabilization of the epidermis–dermis junction [6,7]. It is of note that, compared to the undulating pattern of the basement membrane, the outer surface of the epidermis is much smoother [2]. Indeed, the thickness of the epidermis varies along the interfollicular epidermis; the epidermis in rete ridge regions is thicker than that between the ridges. These observations raise a hypothesis that the keratinocyte population may sense and respond to the bumpy topography of the underlying substrate, thereby forming a cell sheet with a flat outer surface.

Effects of the substrate topography with the scale of tens-to-hundreds μm on keratinocyte behaviors have been investigated using microfabrication techniques. When keratinocytes were grown on the topographically micropatterned substrates, substrates with narrower and deeper wells/channels modulated proliferation and differentiation of keratinocytes, inducing the formation of thicker epidermal layers [8,9,10,11]. These results are consistent with the hypothesis that keratinocytes may have the ability to fill in the substrate undulation, thereby flattening the surface topography of the cell sheet. However, this hypothesis has not been directly tested. Unveiling the properties and mechanisms of how keratinocytes behave on a bumpy substrate to form a cell sheet would contribute to the development of efficient methodologies for skin tissue engineering and regeneration using artificial scaffolds.

In this study, using a microfabricated substrate with a step pattern, we examine whether keratinocytes fill in the substrate topography to form a smooth cell sheet. Furthermore, the roles of cell–cell adhesion and cell proliferation in the formation of a keratinocyte sheet on the stepped substrate are investigated.

## 2. Materials and Methods

### 2.1. Fabrication of the Micropatterned PDMS Substrate

**Figure 1:**
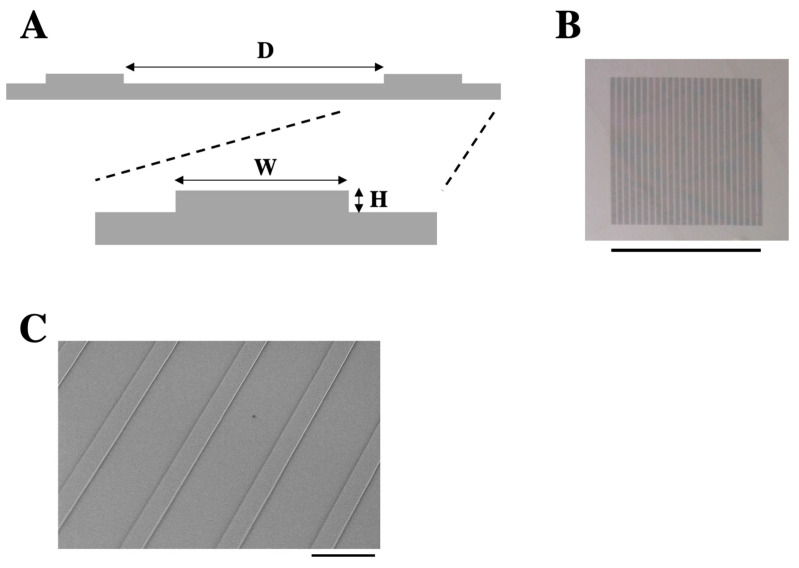
The micropatterned substrate with parallel linear steps. (**A**) Schematic cross-sectional view of the parallel linear steps. H: step height (20 μm), W: step width (150 μm), D: side-to-side distance between steps (500 μm). (**B**) A photograph of the silicon wafer mold. In a 15 × 15 mm region, 20-μm-deep and 150-μm-wide grooves were patterned with 500-μm spacing between them. Scale bar: 15 mm. (**C**) A scanning electron micrograph of the micropatterned substrate. Scale bar: 500 μm.

The polydimethylsiloxane (PDMS) substrate with micropatterns of parallel linear steps (20 μm in height, 150 μm in width, 15 mm in length, and 500 μm in side-to-side distance between steps) (Figure 1A,B) was prepared as described below. Silicon molds were fabricated by deep reactive ion etching (deep RIE). Briefly, a negative photoresist (ZPN1150; Zeon Corporation, Tokyo, Japan) was spin-coated on a wafer and patterned by photolithographic technique. Then, exposed areas were etched by reactive ion deep silicon etching (RIE-800iPB-KU; Samco Inc., Kyoto, Japan) to create a micropattern of linear steps on the wafer. Using the silicon wafer with micropatterned features as a mold (Figure 1B), we created PDMS substrates with the linear step topography. A degassed mixture of the base and the curing agent of Sylgard 184 (Dow Corning, Midland, MI, USA) at the 10:1 ratio was poured onto the silicon wafer mold. After curing at 80 °C for 2 h, the PDMS piece was peeled off and adhered onto a 35-mm glass-bottom dish (AGC Techno Glass, Yoshida, Japan), with the micropatterned surface facing upward.

The surface of micropatterned PDMS was conjugated with collagen, as follows. After applying plasma, the PDMS surface was treated with 1.2% 3-aminopropyltrimethoxysilane (Fujifilm Wako Pure Chemical, Osaka, Japan) in methanol, which was followed by washing with methanol and heating at 60 °C for 30 min. The surface was then treated with 0.5% glutaraldehyde in PBS, washed with PBS, and treated with 50 μg/mL collagen (IPC-50, Koken, Tokyo, Japan). After being washed with and immersed in Dulbecco’s modified Eagle’s medium (DMEM) containing 10% fetal bovine serum (FBS), the collagen-conjugated PDMS was used for cell culture.

### 2.2. Cell Culture

Human HaCaT keratinocytes (Cell Lines Service, Eppelheim, Germany) and HEK293T cells (provided by Dr. Keiko Kawauchi) were maintained in high-glucose DMEM (Nacalai Tesque, Kyoto, Japan) supplemented with 10% FBS (Thermo Fisher Scientific, Waltham, MA, USA).

HaCaT cells expressing short hairpin RNA (shRNA) against α-catenin or non-targeting shRNA were described previously [12]. In brief, these cells were generated using the retrovirus system. The pSUPER.retro.puro vector carrying the sequence of either shRNA against human α-catenin or non-targeting shRNA was co-transfected with the pE-ampho vector into HEK293T cells using the GeneJuice transfection reagent (Merck, Darmstadt, Germany). The target sequences were 5′-GACTTAGGAATCCAGTATA-3′ for human catenin alpha-1 and 5′-ATAGTCACAGACATTAGGT-3′ for non-targeting control. The HEK293T cell culture media containing viral particles were collected at 48 h after the transfection, filtered through 0.45-μm filters, and used for infection into HaCaT cells in the presence of 8 μg/mL polybrene (Sigma-Aldrich, St. Louis, MO, USA). Infected HaCaT cells were selected with 4 μg/mL puromycin (Sigma-Aldrich).

For most experiments, cells were seeded onto micropatterned, collagen-conjugated PDMS substrates at a density of 3.6 × 10^4^ cells/mm^2^ and cultured for 2 days in DMEM supplemented with 10% FBS. When indicated, PF-573228 (10 μM, Sigma-Aldrich), mitomycin C (MMC; 5 μM, Sigma-Aldrich), or blebbistatin (50 μM, Toronto Research Chemicals, Toronto, ON, Canada) was added to the culture medium. Alternatively, for experiments involving low-concentration MMC treatments, cells were seeded onto the substrates at a density of 6.5 × 10^2^ cells/mm^2^ and cultured for 2 days in DMEM containing 10% FBS, which was followed by an additional 2-day culture in the presence of MMC at the indicated concentration.

### 2.3. F-Actin Staining

HaCaT keratinocytes grown on the micropatterned PDMS substrate were fixed and permeabilized for 30 min with 4% formaldehyde and 0.2% Triton X-100 in the cytoskeleton stabilizing buffer (137 mM NaCl, 5 mM KCl, 1.1 mM Na_2_HPO_4_, 0.4 mM KH_2_PO_4_, 4 mM NaHCO_3_, 2 mM MgCl_2_, 5.5 mM glucose, 2 mM EGTA and 5 mM PIPES, pH 6.1) [13], which was followed by blocking with 1% BSA in the cytoskeleton stabilizing buffer for 30 min. The cells were then subjected to filamentous actin (F-actin) staining with Alexa Fluor 546-phalloidin (Thermo Fisher Scientific) for 30 min.

### 2.4. EdU Incorporation Assay

HaCaT keratinocytes grown on the micropatterned PDMS substrate for 2 days in the presence or absence of the indicated drug were incubated with 10 μM EdU (5-ethynyl-2′-deoxyuridine, Thermo Fisher Scientific) at 37 °C for 1 h in the presence or absence of the same drug. These cells were fixed with 4% formaldehyde in PBS for 15 min and permeabilized with 0.5% Triton X-100 in PBS for 20 min. Incorporated EdU was visualized with Alexa Fluor 488-azide using the Click-iT technology (Thermo Fisher Scientific). Total nuclei were stained with 20 μg/mL Hoechst 33342 (Thermo Fisher Scientific).

### 2.5. Confocal Microscopy

Fluorescently labeled cells were observed using a confocal microscope (D-Eclipse C1, Nikon, Tokyo, Japan) equipped with an oil immersion objective (Plan Apo 60XA, NA 1.40, Nikon). 3D confocal Z-stacks were acquired with 0.5-μm intervals. Acquired images were analyzed using the public domain software ImageJ2 (version 2.14.0/1.54f).

In an image sectioned across a linear step of the substrate, the shape of the upper surface of the cell sheet near the substrate step edge was fitted with the logarithmic function,

(1) $$Z=A-B\times \log\left(X-X_{0}\right),$$

where *Z* is the position in the vertical direction, *X* is the horizontal distance from the horizontal position (*X*_0_) of the step edge, and *A* and *B* are constants. Numerical fitting was performed using the KaleidaGraph software (version 5.01, Synergy Software, Reading, PA, USA).

To assess how well the cell sheet filled in the stepped topography, we defined a rectangular region (25 × 45 μm) with its vertex aligned to the lower corner of the step edge in an image sectioned across a linear step of the substrate. Within this region, we quantified the proportion of the area occupied by the cell sheet, hereafter referred to as the ‘cell occupation ratio’ (COR).

### 2.6. Scanning Electron Microscopy

The morphology of the micropatterned PDMS substrate was observed by scanning electron microscopy (SEM). The substrate was subjected to Au coating and then observed with a scanning electron microscope (JSM-5600, JEOL, Akishima, Japan) at an accelerating voltage of 1.5 kV.

### 2.7. Immunoblot

HaCaT keratinocytes confluently cultured on a collagen-coated 35-mm cell culture dish (Corning, Corning, NY, USA) for 2 days were lysed with 2 × lithium dodecyl sulfate sample buffer (Thermo Fisher Scientific) containing 2.5% β-mercaptoethanol. The lysate was resolved by SDS-PAGE (4–12% Bis-Tris gel, Thermo Fisher Scientific), transferred onto the polyvinylidene fluoride membrane (Merck), and probed with primary and Horseradish peroxidase (HRP)-conjugated secondary antibodies. Antibodies used were the rabbit polyclonal antibody against α-catenin (C2081, Sigma-Aldrich), the rabbit monoclonal antibody against E-cadherin (3195, Cell Signaling Technology, Danvers, MA, USA), the mouse monoclonal antibody against β-actin (010-27841, Fujifilm Wako Pure Chemical), the HRP-conjugated anti-rabbit IgG antibody (Medical & Biological Laboratories, Tokyo, Japan), and the HRP-conjugated anti-mouse IgG antibody (Invitrogen, Waltham, MA, USA). Immuno-reactive bands were detected with the SuperSignal West Femto or Pico PLUS chemiluminescent substrate reagent (Thermo Fisher Scientific).

### 2.8. Mechanical Stretching

HaCaT keratinocytes stably expressing shRNA against α-catenin or non-targeting shRNA were seeded onto PDMS-based stretch chambers (SC-0022, Strex, Osaka, Japan) at a density of 3.6 × 10^4^ cells/mm^2^ and cultured for 2 days in DMEM supplemented with 10% FBS. The surface of the stretch chambers was pre-conjugated with collagen using the method described above. The chamber containing a keratinocyte sheet was placed on a motorized stretching device (NS350C, Strex) mounted onto a phase contrast microscope (Lumascope 620, Etaluma, San Diego, CA, USA) that was equipped with a 10× objective (CAch N 10×/0.25 IPC Phase, Olympus, Tokyo, Japan) and settled in a CO_2_ incubator. The chamber was uniaxially stretched by 10%, held for 5 min, and then returned to its original position. Phase contrast images of the keratinocyte sheet during stretching and recovery were captured at 4-s intervals.

A deformation map of the keratinocyte sheet was obtained by particle image velocimetry (PIV) analysis using the ImageJ plugin “PIV” [14]. Two phase contrast images of the keratinocyte sheet captured immediately before the onset of stretching and immediately after the recovery from stretching were used for PIV calculation.

### 2.9. Statistical Analysis

In the quantification graphs, each bar or plot was presented as the mean ± SD. Statistical significance was assessed by Student’s two-tailed, unpaired *t*-test or Welch’s one-way ANOVA followed by Tukey’s post hoc test, using the open-source software Jamovi (version 2.5.2.0).

## 3. Results

**Figure 2:**
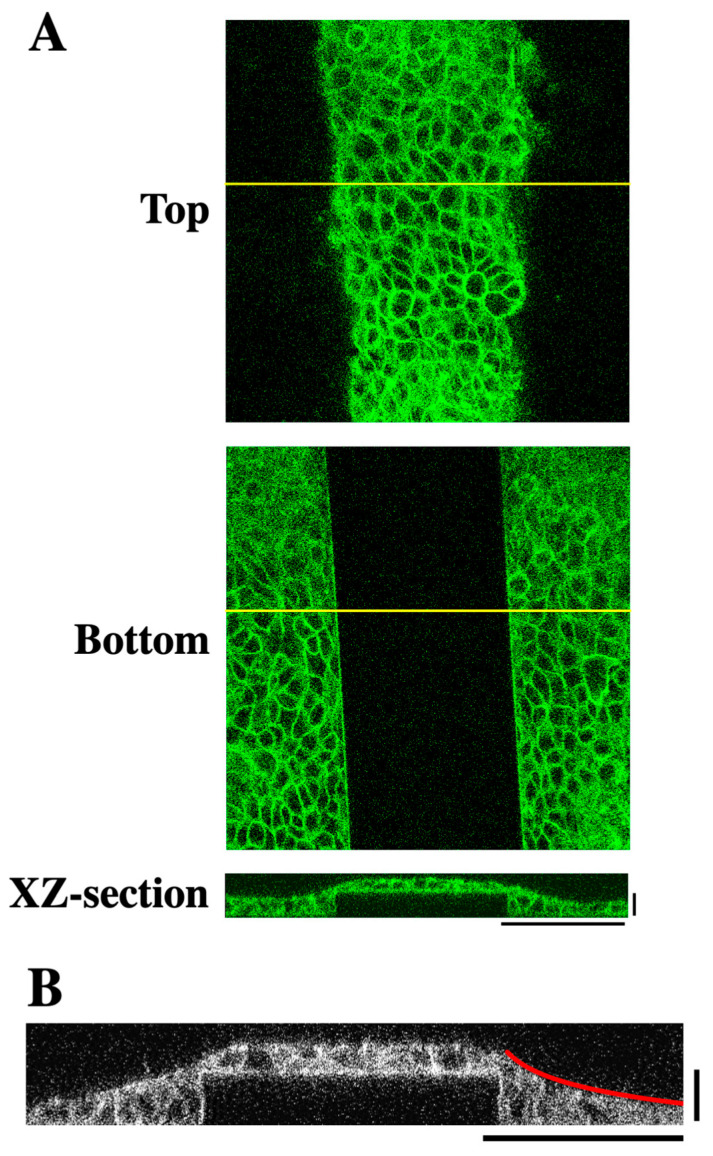
Keratinocytes on the stepped substrate form a sheet with a logarithmically sloped apical surface. (**A**) Confocal micrographs of an F-actin-stained HaCaT cell sheet formed on the linear step substrate. Images at the upper (**Top**) and lower (**Bottom**) levels of the step are shown. An XZ-section image along the yellow lines in the Top and Bottom images is also shown. Horizontal scale bar: 100 μm, vertical scale bar: 20 μm. (**B**) An XZ-section image of an F-actin-stained HaCaT cell sheet formed on the linear step substrate. The apical surface of the cell sheet near the step edge was fitted by a logarithmic curve (red line). Horizontal scale bar: 100 μm, vertical scale bar: 20 μm.

To investigate the effect of substrate topography on the architecture of keratinocyte sheets, we fabricated a PDMS substrate with micropatterned parallel linear steps (20 μm in height, 150 μm in width, 15 mm in length, and 500 μm spacing between steps) (Figure 1A–C). The surface of the substrate was conjugated with collagen to enable cell adhesion to the substrate. Human HaCaT keratinocytes, which were seeded onto the micropatterned substrate at a confluent cell density and grown for 2 days, were stained for F-actin and observed using a confocal microscope. The cells covered the entire surface of the substrate including the lateral faces of each step (Figure 2A). The step height used in this study (i.e., 20 μm) corresponded to the height of approximately two to three cells (Figure 2A,B). XZ-section images across the linear step showed that the surface of the cell sheet near the step edge did not exhibit a step-like profile but instead exhibited a sloped topography (Figure 2A,B). This slope was well approximated by a logarithmic curve (Figure 2B and Figure S1). These results reveal that keratinocytes fill in the stepped topography of the substrate, forming a cell sheet with a smooth surface.

The logarithmic curvature of the cell sheet surface near the step edge implicates elastic deformation of the cell sheet, as the deformation of an elastic membrane under a localized perpendicular force typically assumes a logarithmic profile [15,16]. Indeed, keratinocyte sheets have been shown to behave as elastic membranes [16]. Intercellular adherens junctions (AJs) and actomyosin contractility reportedly contribute to elastic properties of epithelial cell sheets [16,17]. Therefore, we next examined the roles of adherens junctions and actomyosin contractility in the formation of keratinocyte sheets with a logarithmically sloped apical surface.

**Figure 3:**
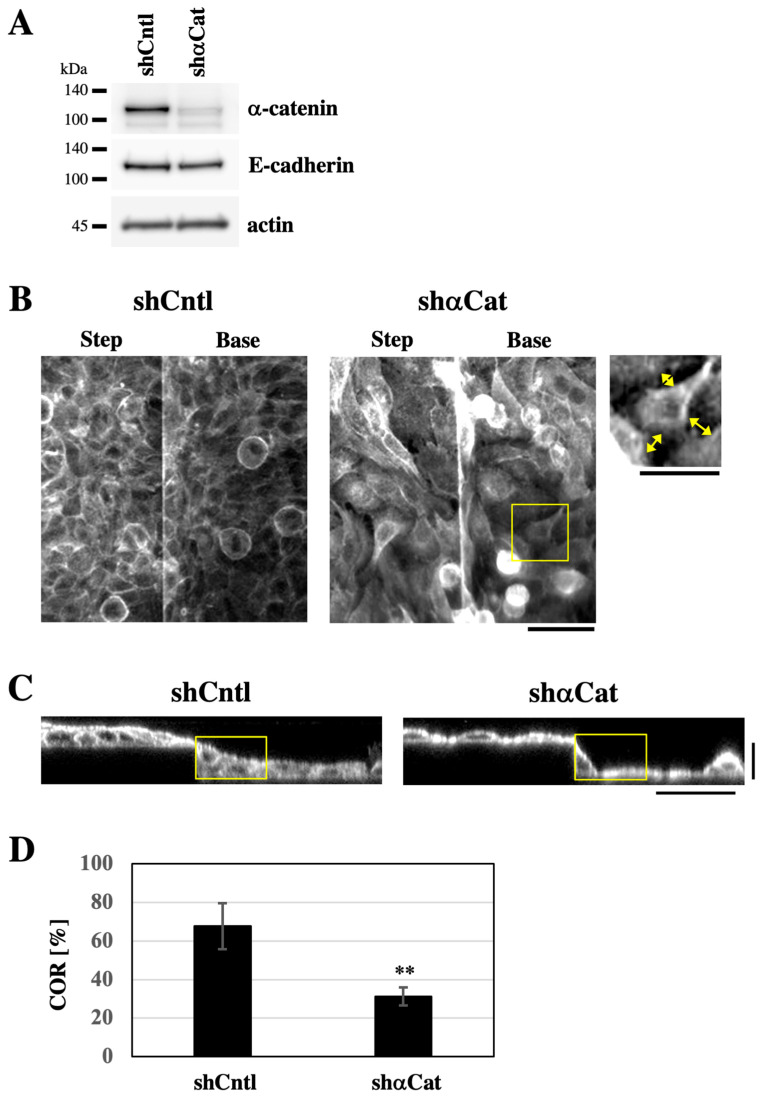
α-Catenin-mediated AJ stability is essential for the formation of a keratinocyte sheet with a smooth apical surface on the stepped substrate. (**A**) Lysates of HaCaT keratinocytes stably expressing shRNA targeting α-catenin (shαCat) or non-targeting control shRNA (shCntl) were subjected to immunoblotting for α-catenin, E-cadherin, and actin. (**B**) F-actin-stained HaCaT cell sheets, which stably expressed either shCntl or shαCat and formed on the linear step substrate, were imaged using confocal microscopy. Projected Z-stack images covering multiple focal planes from the upper to lower levels of the step are shown. Each projected image includes both the step and base regions of the substrate. An enlarged view of the yellow-boxed region in the shαCat panel is also shown. Yellow double-headed arrows in the enlarged image indicate intercellular gaps at cell–cell boundaries. Scale bars: 50 μm (projected images), 30 μm (enlarged image). (**C**) XZ-section images of F-actin-stained HaCaT cell sheets stably expressing either shCntl or shαCat and formed on the linear step substrate. Yellow boxes indicate the regions used for analysis of the cell occupation ratio (COR). Horizontal scale bar: 50 μm, vertical scale bar: 20 μm. (**D**) Cell occupation ratio (COR) of HaCaT cell sheets stably expressing either shCntl or shαCat and formed on the linear step substrate. Each bar represents the mean ± SD (*n* = 6). ** *p* < 0.001 (Student’s two-tailed, unpaired *t*-test).

The expression of the AJ component α-catenin was depleted using shRNA to destabilize AJs (Figure 3A) [16,18]. When keratinocyte sheets expressing either shRNA targeting α-catenin or non-targeting control shRNA, which were grown on PDMS-based stretch chambers, were subjected to one cycle of uniaxial stretching and recovery, the α-catenin-depleted sheets retained significantly greater residual deformation after recovery from stretching than the control sheets (Figure S2, Videos S1 and S2), suggesting that α-catenin depletion makes cell sheets less elastic. When grown on the substrate with the stepped topography, both α-catenin-depleted cells and control shRNA-expressing cells covered the entire surface of the substrate (Figure 3B). Notably, α-catenin-depleted cells exhibited intercellular gaps at cell–cell boundaries, indicating destabilization of AJs (Figure 3B). In XZ-section images, cells expressing control shRNA formed sheets with smoothly sloping surface topography around step edges (Figure 3C and Figure S3). By contrast, α-catenin-depleted cell sheets displayed steep changes in surface slope around step edges (Figure 3C and Figure S3). To assess how well the cell sheet filled in the stepped topography, we defined a rectangular region (25 × 45 μm) with its vertex aligned to the lower corner of the step edge (Figure 3C, yellow rectangles). Within this region, we quantified the proportion of the area occupied by the cell sheet, hereafter referred to as the ‘cell occupation ratio’ (COR). The COR value was significantly lower in α-catenin-depleted cells compared to control shRNA-expressing cells (Figure 3D). These results suggest that α-catenin-mediated AJ stability is essential for forming keratinocyte sheets with a smooth apical surface on the stepped substrate.

To suppress the actomyosin contractility, HaCaT keratinocytes were seeded and grown for 2 days in the presence of the myosin II ATPase inhibitor blebbistatin. Treatment with 50 μM blebbistatin, which reportedly suppressed actomyosin contraction in HaCaT cell sheets [16,19], did not alter the surface topography of the cell sheet grown on the step-patterned substrate (Figure S4A). The COR value was not significantly affected by the blebbistatin treatment (Figure S4B), suggesting that the myosin II-generated contractile force was not required for the formation of smooth keratinocyte sheets on the substrate with stepped topography.

**Figure 4:**
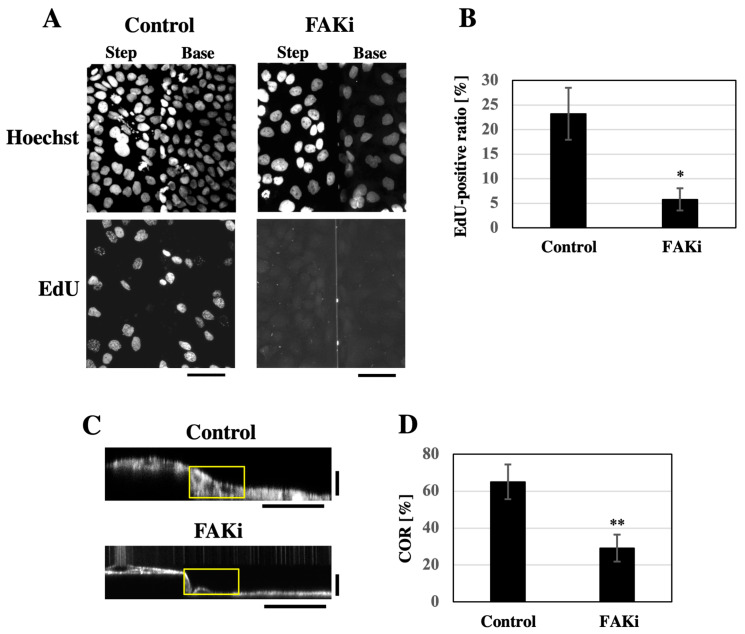
FAK inhibition impairs the formation of a keratinocyte sheet with a smooth apical surface on the stepped substrate. (**A**) EdU was incorporated into HaCaT cell sheets formed in the presence of either 10 μM PF-573228 (FAK inhibitor; FAKi) or DMSO (Control) on the linear step substrate. Total nuclei were counterstained with Hoechst. Projected Z-stack images covering multiple focal planes from the upper to lower levels of the step are shown. Each projected image includes both the step and base regions of the substrate. Scale bars: 50 μm. (**B**) Ratio of EdU-positive cells in HaCaT cell sheets formed in the presence of either 10 μM PF-573228 (FAKi) or DMSO (Control). Each bar represents the mean ± SD (*n* = 6). * *p* < 0.05 (Student’s two-tailed, unpaired *t*-test). (**C**) XZ-section images of F-actin-stained HaCaT cell sheets formed in the presence of either 10 μM PF-573228 (FAKi) or DMSO (Control) on the linear step substrate. Yellow boxes indicate the regions used for analysis of the cell occupation ratio (COR). Horizontal scale bars: 50 μm, vertical scale bars: 20 μm. (**D**) Cell occupation ratio (COR) of HaCaT cell sheets formed in the presence of either 10 μM PF-573228 (FAKi) or DMSO (Control). Each bar represents the mean ± SD (*n* = 6). ** *p* < 0.001 (Student’s two-tailed, unpaired *t*-test).

Micropatterned substrates have been shown to promote the proliferation of keratinocytes compared to flat substrates [11,20]. We next asked whether cell proliferation contributed to the formation of smooth keratinocyte sheets on the substrate with stepped topography. Focal adhesion kinase (FAK), a non-receptor tyrosine kinase, is involved in the proliferation of various cell types, including keratinocytes [21,22]. HaCaT keratinocytes were seeded onto the stepped substrate at a confluent cell density and grown for 2 days in the presence of the FAK inhibitor PF-573228 [23]. Treatment with the FAK inhibitor caused a decrease in the cell density within the keratinocyte sheets (Figure 4A). When the proliferative ability of cells was assessed by nuclear incorporation of EdU, the proportion of EdU-positive cells was markedly lower in FAK-inhibited cells than in control cells (Figure 4A,B), indicating that FAK inhibition suppressed keratinocyte proliferation. Correspondingly, under FAK inhibition, the keratinocyte sheets filled in the stepped topography of the substrate to a lesser extent than the control sheets (Figure 4C and Figure S5). The COR value was significantly lower in FAK-inhibited cells compared to control cells (Figure 4D).

**Figure 5:**
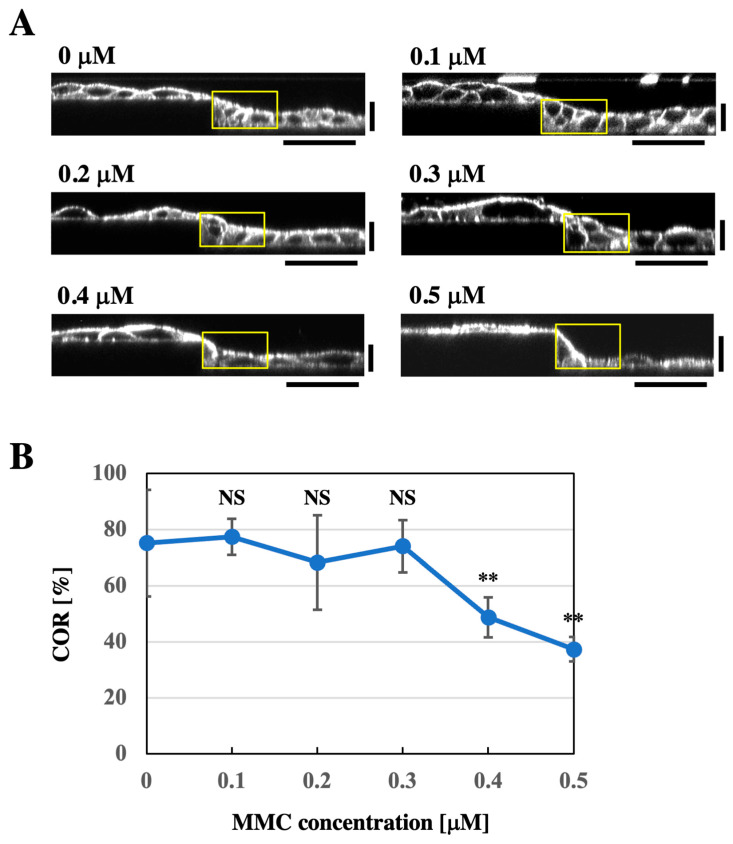
Mitomycin C (MMC) treatment impairs the formation of a keratinocyte sheet with a smooth apical surface on the stepped substrate. (**A**) XZ-section images of F-actin-stained HaCaT cell sheets formed in the presence of various concentrations of MMC on the linear step substrate. MMC was applied two days after seeding cells onto the stepped substrate at a low cell density, and the cells were cultured for additional two days in the presence of MMC. Yellow boxes indicate the regions used for analysis of the cell occupation ratio (COR). Horizontal scale bars: 50 μm, vertical scale bars: 20 μm. (**B**) Cell occupation ratio (COR) of HaCaT cell sheets formed in the presence of different concentrations of MMC. Each plot represents the mean ± SD (*n* = 6). ** *p* < 0.01, NS: not significantly different from the 0 μM control (Welch’s one-way ANOVA followed by Tukey’s post hoc test).

To further investigate the role of cell proliferation in the formation of logarithmically sloped keratinocyte sheets on the substrate with the stepped topography, we used mitomycin C (MMC), an antiproliferative agent that inhibits DNA synthesis. The addition of MMC (up to 5 μM) to the culture medium during seeding and growing cells at a confluent cell density for 2 days did not impair the formation of cell sheets with a smooth apical surface (Figure S6). This result may reflect the previous finding that MMC does not arrest cell proliferation under confluent cell density conditions [24]. To overcome this limitation of MMC usage, we applied MMC to cells grown for 2 days at a subconfluent density on the micropatterned substrate, and then the cells were further grown for additional 2 days in the presence of MMC. Notably, this modification to the cell culture protocol alone (i.e., without MMC addition) did not affect the formation of keratinocyte sheets that filled in the stepped topography of the substrate and maintained the smooth apical surface (Figure 5A and Figure S7, 0 μM). However, when 0.4 or 0.5 μM MMC was added under this modified protocol, the upper surface of the cell sheets became less smooth, exhibiting steep changes in slope around step edges of the substrate (Figure 5A and Figure S7). While the treatment with 0.1–0.3 μM MMC did not alter the COR value, the treatment with 0.4 or 0.5 μM MMC significantly reduced it (Figure 5B).

**Figure 6:**
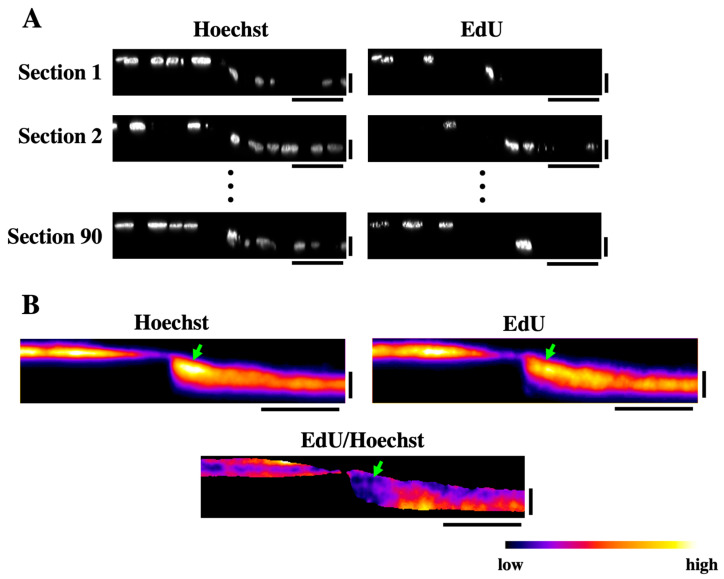
Spatial distribution of proliferative cells on the stepped substrate. (**A**) EdU was incorporated into HaCaT cell sheets formed on the linear step substrate. Total nuclei were counterstained with Hoechst. Multiple XZ-section images of EdU and Hoechst (90 sections each) were acquired. Horizontal scale bars: 50 μm, vertical scale bars: 20 μm. (**B**) Acquired XZ-section images of EdU and Hoechst (90 sections each) were averaged. The ratio image of the averaged EdU image against the averaged Hoechst image is also shown. Green arrows indicate the region near the step edge, where strong Hoechst signal and low EdU/Hoechst ratios were observed. Horizontal scale bars: 50 μm, vertical scale bars: 20 μm.

The results of FAK inhibition and MMC treatment strongly suggest that the formation of logarithmically sloped cell sheets on the stepped substrate depends on cell proliferation. As a hypothesis for the underlying mechanism, cell proliferation may be promoted in the region near the step edge to fill in the stepped topography of the substrate. To test this hypothesis, we examined the distribution of EdU-positive proliferating cells on the stepped substrate. Numbers of XZ-section images of Hoechst-stained total nuclei and EdU-positive nuclei (90 images each) were acquired (Figure 6A), and these images were, respectively, averaged (Figure 6B, ‘Hoechst’ and ‘EdU’). In the averaged image of Hoechst-stained nuclei, a high signal intensity was observed near the rising edge of the step (Figure 6B, green arrow in ‘Hoechst’), indicating a relatively high cell density in this region. However, the ratio image of the averaged EdU signal against the averaged Hoechst signal revealed that the relative intensity of EdU against Hoechst was low near the step edge (Figure 6B, green arrow in ‘EdU/Hoechst’), suggesting that the relative activity of cell proliferation was low in this region. From these results, it was suggested that cells accumulated preferentially in the regions near the rising edges of the steps. Still, this accumulation was not attributable to locally increased cell proliferation.

## 4. Discussion

Morphogenesis of the epidermis depends on both cell–cell adhesion and cell proliferation. The stratified architecture of the epidermis, which relies on intercellular adhesions within and between cell layers [1], is maintained through continuous epidermal turnover. Basal cells adhering to the basement membrane proliferate to produce daughter cells that differentiate, move to upper layers, and eventually desquamate from the outermost surface, driving the renewal process [25]. However, the mechanism underlying the homeostasis of the epidermal morphology remains unclear. How does the epidermis in interfollicular regions maintain a flat outer surface despite the undulating topography of the underlying basement membrane? In this study, using a substrate with micropatterned steps of a few cell heights, we revealed for the first time that keratinocytes on the topographically patterned substrate autonomously form a cell sheet with a smooth upper surface. Keratinocytes tended to accumulate near the rising edge of a step, creating a sheet characterized by a logarithmic slope on its upper surface. We further found that both AJ-mediated cell–cell adhesion and cell proliferation were essential for the formation of this smooth cell sheet. In our experimental model, keratinocytes on the stepped substrate are proliferative in the nutrient-rich liquid environment, which mimics the basal layers of the epidermis. Therefore, our results in this study imply that basal keratinocytes may contribute to resolving the topographical mismatch between the undulating basement membrane and the flat upper layers, including the stratum granulosum and stratum corneum, in the epidermis.

In the case of a suspended 2D keratinocyte sheet, a logarithmic curvature of the sheet edge is formed by the balance between a local outward-pulling force acting perpendicular to the edge and the elastic tension within the sheet [16]. Logarithmic out-of-plane deformation of an elastic membrane is also caused by a balance between a local force perpendicular to the membrane and the elastic membrane tension [15]. In our experimental model of this study, the logarithmic curvature of the keratinocyte sheet surface near the step edge of the substrate potentially represents the balance between the force acting on the sheet at the step corner and the elastic tension developed in the sheet. We found that the formation of the logarithmically curved surface was impaired upon α-catenin depletion, which may be, at least in part, due to a reduction in the elasticity of the cell sheet, as destabilization of AJs by α-catenin depletion has been reported to fluidize keratinocyte sheets [16,18].

Myosin II-mediated contractility is also known to contribute to the elasticity of epithelial cell sheets, including keratinocyte sheets [16,17]. However, myosin II inhibition by blebbistatin treatment did not affect the surface topography of keratinocyte sheets in this study. It was reported that inhibition of myosin II decreased the elasticity of the epithelial cell sheet by 36% [17]. The residual elasticity independent of myosin II may be sufficient for the formation of the logarithmically curved surface in keratinocyte sheets on our stepped substrate.

We revealed that cell proliferation is essential for filling in the stepped topography of the substrate to form a keratinocyte sheet with a smooth apical surface. Recent studies have shown that the local curvature of the substrate influences cell proliferation, even though different cell types likely exhibit different dependencies on the substrate curvature. On one hand, cell proliferation in epithelial cell sheets is attenuated on substrates with negative curvatures compared to flat or positively curved substrates [26]. On the other hand, intestinal stem cells are preferentially proliferative in regions of large negative curvatures such as crypts [27]. If proliferation of keratinocytes was promoted around lower corners of step edges (i.e., regions with infinitely negative curvatures), the keratinocyte sheet would be thickened in these regions, smoothening the surface of the sheet formed on the stepped substrate. However, this scenario was not the case, because our results showed that cell proliferation was not promoted, but rather attenuated, around the lower corners. Nevertheless, we found that the cell density in the keratinocyte sheet was relatively high around the lower corners of step edges. These results suggest that cells accumulate in the regions around the lower corners without locally promoting cell proliferation in these regions.

Currently, it is unclear how keratinocytes accumulate in the regions around the lower corners of step edges. However, cell movement towards these regions may contribute to this process. FAK promotes both the proliferation and migration of various cell types, including keratinocytes [21,22,28,29]. In this study, we found that FAK inhibition with PF-573228 abrogated the formation of a smooth keratinocyte sheet on the step-patterned substrate. This effect of FAK inhibition may result from the suppression of not only cell proliferation but also cell migration. The precise role of cell migration in the formation of a smooth keratinocyte sheet on the topographical substrate needs to be examined in future studies.

## Data Availability

The data that support the findings of this study are available from the corresponding author.

## Supplementary Materials

The following supporting information can be downloaded at: https://www.mdpi.com/article/10.3390/bioengineering12090929/s1, Figure S1: Logarithmic curve fitting of the apical surface slope of HaCaT cell sheets. Figure S2: Residual deformation of control and α-catenin-depleted HaCaT cell sheets after one cycle of stretching and recovery. Figure S3: Enlarged images of the step edge regions in Figure 3C. Figure S4: Myosin II inhibition with blebbistatin does not affect the formation of a keratinocyte sheet on the stepped substrate. Figure S5: Enlarged images of the step edge regions in Figure 4C. Figure S6: Culturing cells at a confluent density in the presence of mitomycin C (MMC) does not impair the formation of a keratinocyte sheet with a smooth apical surface on the stepped substrate. Figure S7: Enlarged images of the step edge regions in Figure 5A. Video S1: A phase contrast movie of an α-catenin-depleted keratinocyte sheet during stretching and recovery. Video S2: A phase contrast movie of a control keratinocyte sheet during stretching and recovery.
