# Supplementary material for: Keratinocyte Capability for Smooth Sheet Formation on a Step Pattern Substrate
Source: Bioengineering (Basel). 2025 Aug 29;12(9):929. doi: 10.3390/bioengineering12090929 (PMC12467466; doi:10.3390/bioengineering12090929)
Supplement: Supplementary file 1 [file bioengineering-12-00929-s001.zip › Supplementary.pdf]

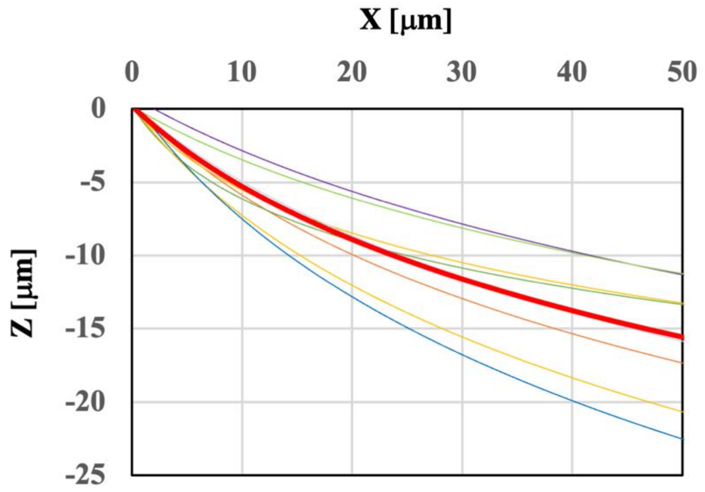

Figure S1. Logarithmic curve fitting of the apical surface slope of HaCaT cell sheets. In XZ-section images of F-actin-stained HaCaT cell sheets on the linear step substrate, the apical surface slope profiles around step edges were fitted with logarithmic curves using Equation [1] in Materials and Methods. Thin lines represent fitted curves for individual sheets (eight in total), while the thick red line represents a logarithmic curve based on Equation [1], using the average values of A, B, and X_0 from the eight fitted results. The zero point in Z for each curve was set to the Z position of the cell sheet surface located above the upper corner of a step.

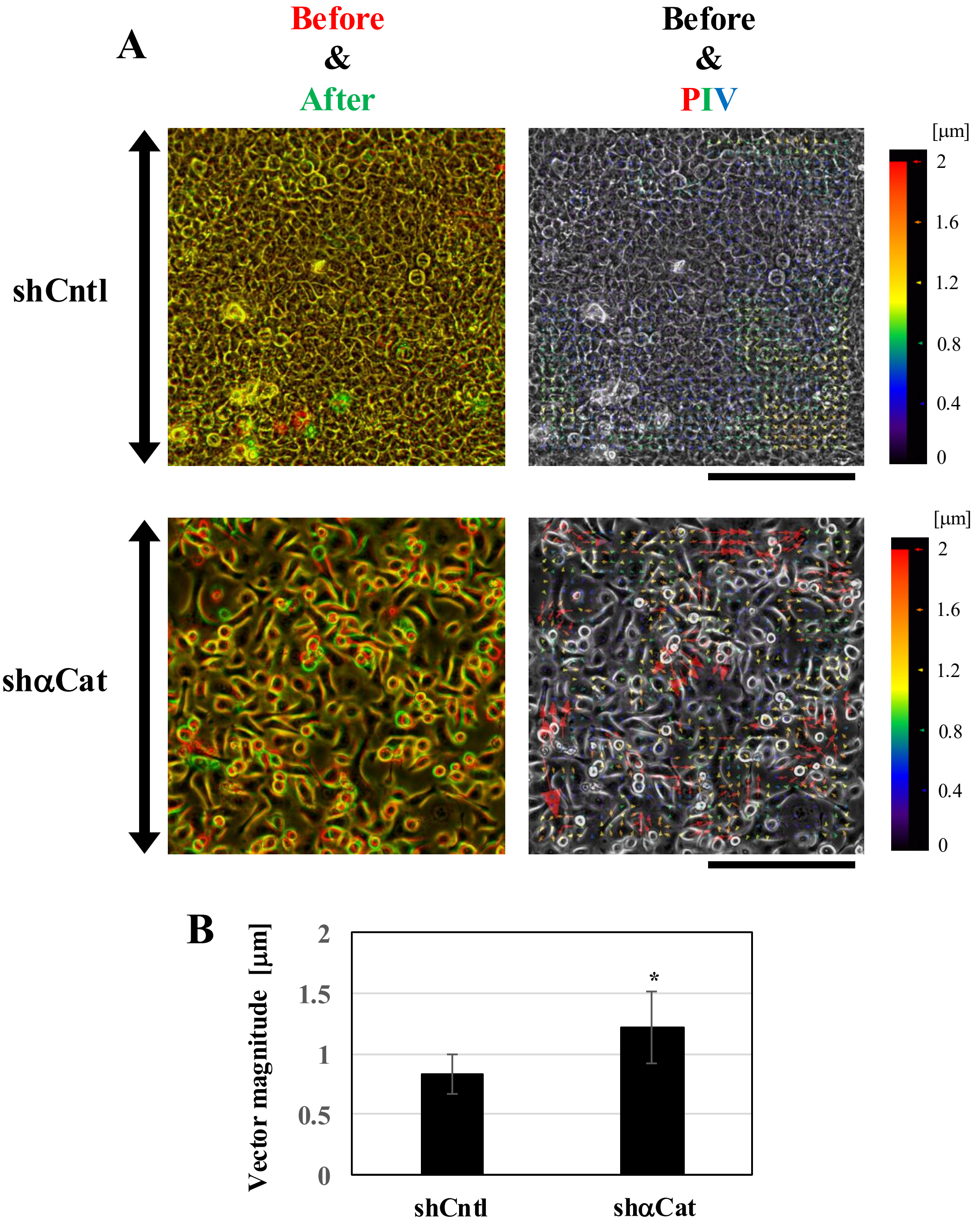

Figure S2. Residual deformation of control and α-catenin-depleted HaCaT cell sheets after one cycle of stretching and recovery. (A) HaCaT cell sheets stably expressing shRNA targeting α-catenin (shαCat) or non-targeting control shRNA (shCntl), which were grown on stretch chambers, were uniaxially stretched by 10%, held for 5 min, and then returned to the original position. Left panels: Phase contrast images of the cell sheets after stretching (green) were overlaid onto those before stretching (red). Double-headed arrows indicate the stretching direction. Right panels: Deformation maps between before and after stretching were overlaid onto phase contrast images of the cell sheets before stretching. Scale bars: 200 μm. (B) The deformation vector magnitude averaged within each deformation map. Each bar represents the mean ± SD (n = 4). *$p < 0.05$ (Student's two-tailed, unpaired *t*-test).

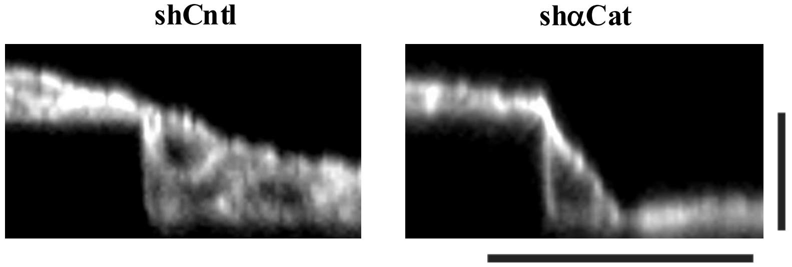

Figure S3. Enlarged images of the step edge regions in Figure 3C. XZ-section images of F-actin-stained HaCaT cell sheets stably expressing shRNA targeting α-catenin (shαCat) or non-targeting control shRNA (shCntl) and formed on the linear step substrate. Horizontal scale bar: 50 μm, vertical scale bar: 20 μm.

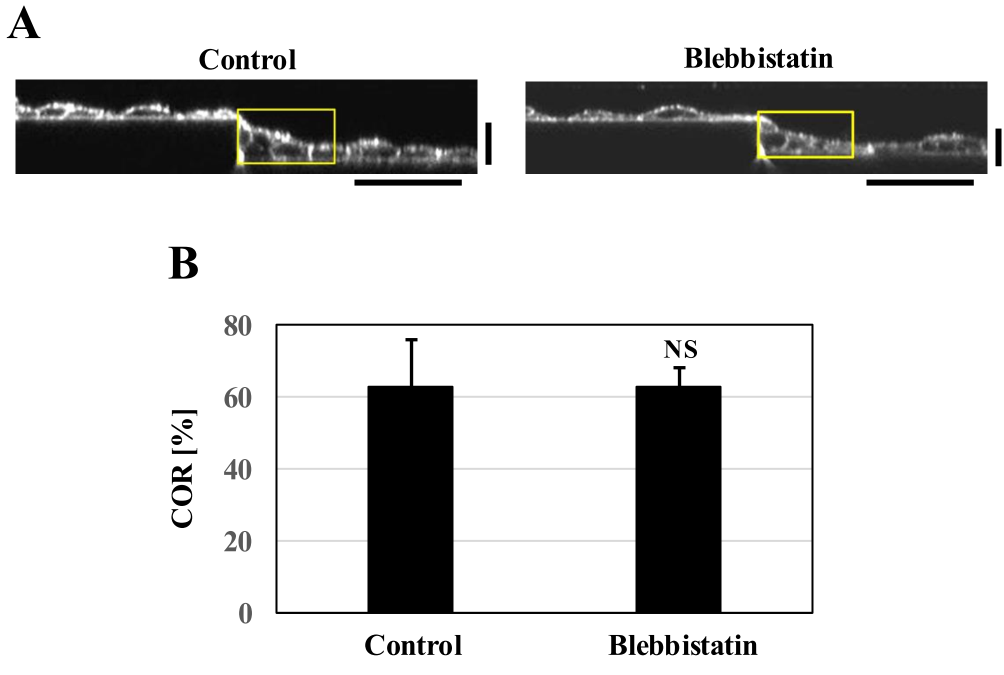

Figure S4. Myosin II inhibition with blebbistatin does not affect the formation of a keratinocyte sheet on the stepped substrate. (A) XZ-section images of F-actin-stained HaCaT cell sheets formed in the presence of either 50 μM blebbistatin or DMSO (Control) on the linear step substrate. Yellow boxes indicate the regions used for analysis of the cell occupation ratio (COR). Horizontal scale bars: 50 μm, vertical scale bars: 20 μm. (B) Cell occupation ratio (COR) of HaCaT cell sheets formed in the presence of either 50 μM blebbistatin or DMSO (Control). Each bar represents the mean ± SD (n = 6). NS: no significant difference (Student's two-tailed, unpaired *t*-test).

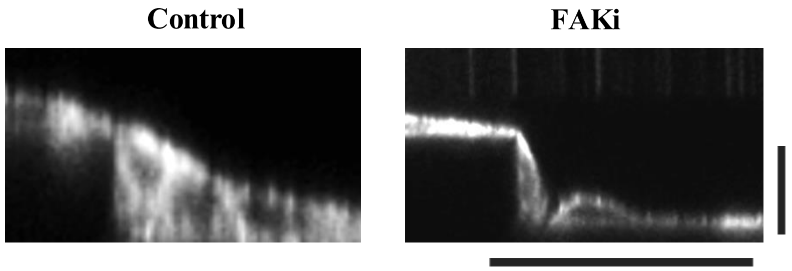

Figure S5. Enlarged images of the step edge regions in Figure 4C. XZ-section images of F-actin-stained HaCaT cell sheets formed in the presence of either 10 µM PF-573228 (FAK inhibitor; FAKi) or DMSO (Control) on the linear step substrate. Horizontal scale bar: 50 µm, vertical scale bar: 20 µm.

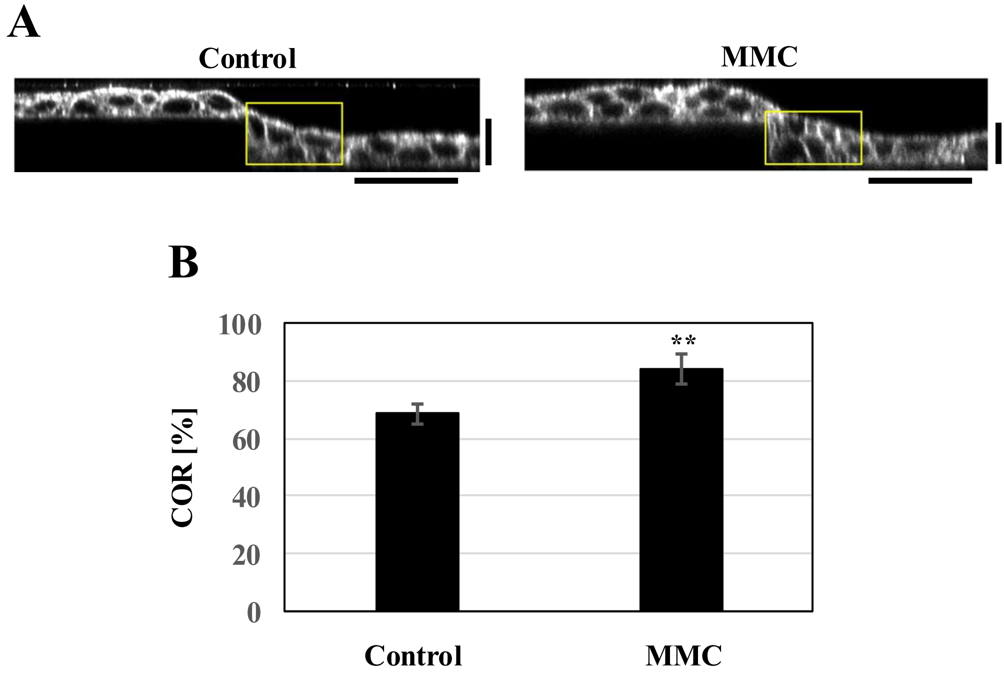

Figure S6. Culturing cells at a confluent density in the presence of mitomycin C (MMC) does not impair the formation of a keratinocyte sheet with a smooth apical surface on the stepped substrate. (A) XZ-section images of F-actin-stained HaCaT cell sheets formed in the presence or absence of 5 µM MMC on the linear step substrate. Cells were seeded at a confluent density onto the stepped substrate and cultured for 2 days with or without MMC. Yellow boxes indicate the regions used for analysis of the cell occupation ratio (COR). Horizontal scale bars: 50 µm, vertical scale bars: 20 µm. (B) Cell occupation ratio (COR) of HaCaT cell sheets formed in the presence or absence of 5 µM MMC. Each bar represents the mean ± SD (n = 6). **$p < 0.001$ (Student's two-tailed, unpaired *t*-test).

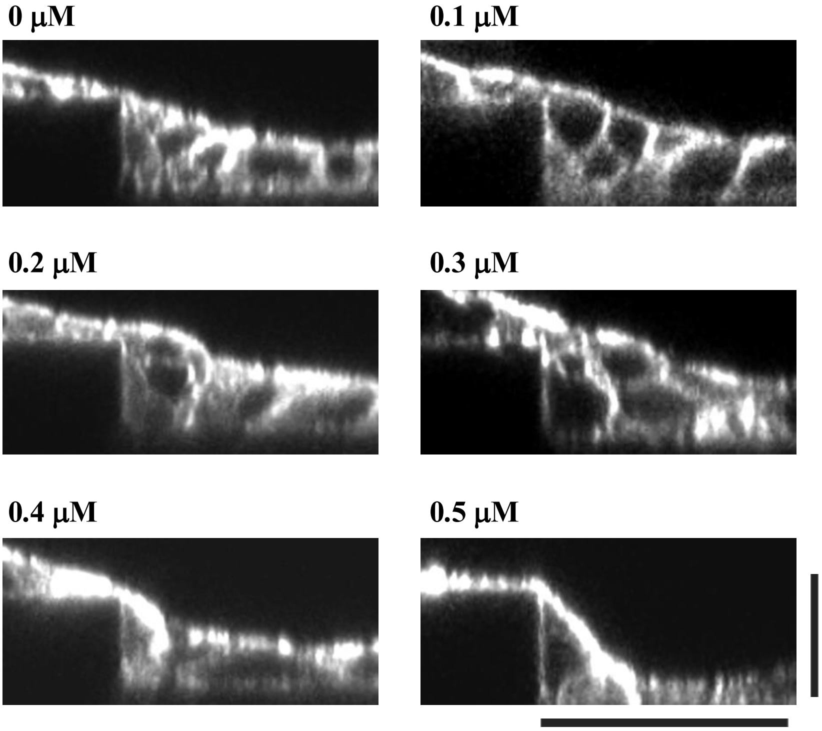

Figure S7. Enlarged images of the step edge regions in Figure 5A. XZ-section images of F-actin-stained HaCaT cell sheets formed in the presence of various concentrations of mitomycin C (MMC) on the linear step substrate. Horizontal scale bar: 50 μm, vertical scale bar: 20 μm.

Video S1. A phase contrast movie of a control keratinocyte sheet during stretching and recovery. A HaCaT keratinocyte sheet expressing non-targeting control shRNA, which was grown on a PDMS-based stretch chamber, was uniaxially stretched by 10%, held for 5 min, and then returned to the original position. Phase contrast images during stretching and recovery, which were captured at 4-s intervals, are shown at the speed of 20 frames per second. An green arrow indicates the stretching direction.

Video S2. A phase contrast movie of a α-catenin-depleted keratinocyte sheet during stretching and recovery. A HaCaT keratinocyte sheet expressing shRNA targeting α-catenin, which was grown on a PDMS-based stretch chamber, was uniaxially stretched by 10%, held for 5 min, and then returned to the original position. Phase contrast images during stretching and recovery, which were captured at 4-s intervals, are shown at the speed of 20 frames per second. An green arrow indicates the stretching direction.
